# Comparison of NK-1 Receptor Antagonist (Maropitant) to Morphine as a Pre-Anaesthetic Agent for Canine Ovariohysterectomy

**DOI:** 10.1371/journal.pone.0140734

**Published:** 2015-10-29

**Authors:** Megan Marquez, Pedro Boscan, Heather Weir, Pamela Vogel, David C. Twedt

**Affiliations:** Department of Clinical Sciences, Veterinary Teaching Hospital, Colorado State University, Fort Collins, Colorado, United States of America; Central South University, CHINA

## Abstract

**Objective:**

To compare the NK-1 receptor antagonist maropitant to morphine during and after surgery in dogs undergoing ovariohysterectomy (OHE).

**Methods:**

30 healthy female dogs were randomly divided to receive either a pre-anaesthetic dose of morphine (0.5 mg/kg SQ) or maropitant (1 mg/kg, SQ) prior to OHE. Anaesthesia was induced with propofol and maintained with isoflurane. Expired isoflurane concentration, heart rate (HR), systolic arterial pressure (SAP) and respiratory rate were measured. Post-operative pain scores and appetite were evaluated during the recovery period. Rescue analgesia (morphine 0.1 mg/kg IV) was administered as needed post-operatively based on blinded pain score assessments.

**Results:**

Although clinically comparable; during surgical stimulation, the maropitant group had lower HR (108±18 vs 115±24 bpm; p = 0.04), lower SAP (114±23 vs 125±23 mmHg; p = 0.003) and required slightly lower percent of isoflurane anaesthetic (1.35±0.2 vs 1.51±0.4%; p = 0.005), when compared to the morphine group. In the recovery period, the maropitant group had lower pain scores at extubation (1.7±0.7 vs 3.4±2.3; p = 0.0001) and were more likely to eat within 3 hours after extubation (64.7 vs 15.3%). However, post-operative rescue analgesia requirements were similar between groups. All other measured parameters were similar between groups. The overall difference observed between groups was small and all monitored and measured parameters were within the expected range for anesthetized dogs.

**Clinical Significance:**

No major differences in cardiorespiratory parameters or anaesthetic requirements were observed between maropitant and morphine when used as a pre-anesthetic agent for OHE. Further studies are necessary to fully elucidate the benefits of maropitant as a pre-anaesthetic agent for canine OHE.

## Introduction

Substance P is a neuropeptide that activates nociceptive pathways associated with pain perception. Substance P activation of pain pathways is mediated through neurokinin 1 (NK-1) receptors. Research using NK-1 receptor antagonists to block nociceptive pathways suggest that NK-1 antagonists may be effective when managing visceral pain in a number of animal studies [1–6]. However, in clinical human trials NK-1 antagonist drugs used to manage and treat nociception or pain, especially somatic pain, have been disappointing [7,8]. The explanation of why NK-1 antagonists may work in some pain models but not in others is unknown. A potential difference may result from the relative density of NK-1 receptors or the amount of substance P released in somatic or visceral afferent pathways [9].

Maropitant (Cerenia; Zoetis) is an NK-1 receptor antagonist approved for use as an antiemetic for dogs and cats [10–13]. The antiemetic effect of maropitant appears to be predominately mediated at the level of the central nervous system. In two studies, maropitant was shown to decrease the inhaled anaesthetic minimum alveolar concentration (MAC) when applying noxious stimulation to the ovary and ovarian ligament in both the dog and the cat [14–15]. Using the recommended antiemetic dose of 1 mg/kg, maropitant decreased the sevoflurane MAC by 24% in dogs and 15% in cats. The degree of visceral stimulation in this model was thought to be similar to the pain sensation associated with ovarian ligament manipulation occurring during standard ovariohysterectomy (OHE) surgery [16]. Consequently, we sought to determine if maropitant, a NK-1 receptor antagonist, improves the anaesthesia quality during OHE. The objective of this clinical study was to compare the anaesthetic and post-operative effects of maropitant compared to morphine, as a standard pre-anaesthetic agent, given to dogs undergoing laparotomy for OHE.

## Materials and Methods

Healthy intact female dogs, presenting to the Veterinary Teaching Hospital (VTH) at Colorado State University (CSU) for elective OHE were considered for the prospective randomized blinded clinical trial. The study was explained to the dog owners. The owners signed a consent form to include their dog in the study. The study was approved by the CSU Institutional Animal Care and Use Committee (protocol number 10-1935A).

Prior to admission into the study, a complete medical history, physical examination, body weight, baseline packed cell volume and total protein were obtained to determine the dog’s health. If all measured parameters were within normal limits and the dog deemed healthy, the dog was included in the study.

A standardized OHE was performed on all dogs by trained 4^th^ year veterinary students under direct supervision of an experienced veterinarian. The OHE surgery protocol followed the CSU-VTH guidelines for an OHE.

Dogs were randomly divided to receive either a pre-anaesthetic dose of morphine sulphate (0.5 mg/kg SQ) or maropitant (1 mg/kg SQ). The pre-anaesthetic medications were administered by a non-blinded investigator approximately 30 minutes before anaesthesia induction. The remainder of the anaesthesia, surgical and postoperative protocol was identical between groups.

Anaesthesia was induced with intravenous propofol (Propoflo; Abbott) to effect and anaesthesia was maintained with isoflurane in oxygen (O_2_) using a precision vaporizer (Penlon). The O_2_ flow during anaesthesia was maintained at 2 L/min using a rebreathing circle system and the rebreathing bag was emptied every time the isoflurane vaporizer was changed. Intravenous fluids (Lactated Ringer’s Solution; Abbott Laboratories) were administered at 5 ml/kg/h throughout the procedure. Variables evaluated during anaesthesia included respiratory rate (RR), expired isoflurane concentration (Ohmeda 5330), heart rate (HR) and rhythm (Multiparameter Physiologic Monitor; Digicare), systolic arterial blood pressure (SAP) using an ultrasonic doppler flow probe (Model 811-B; Parks Medical Electronics, Inc.), as well as all observed clinical responses during surgical stimulation. All variables and observations were recorded by 2 investigators blinded to the treatment group.

The urinary bladder was expressed before the beginning of surgery to minimize any behavioural response to bladder distension during the post-operative period. Laparotomy for OHE was performed via a midline incision caudal to the umbilicus. OHE was performed following the standard guidelines and protocol from the VTH at CSU.

Baseline values were recorded during anaesthesia prior to the beginning of surgery. Values obtained during surgery were recorded at six designated time points; during abdominal cavity incision, while breaking down each ovarian ligament, during uterine body ligation, while closing the abdominal wall and during skin closure. The data obtained during surgery were averaged for analysis.

Following induction of anaesthesia, approximately fifteen minutes prior to surgery, the isoflurane vaporizer was set to 1% expired isoflurane concentration. During surgery the vaporizer setting was adjusted according to the dog’s response to surgical stimulation. A response to surgical stimulation was considered when either: RR, HR and/or SAP increased more than 20% from baseline values or if animal movement was observed. If the dog did not respond to surgical stimulation during the previous 10 minutes, the isoflurane vaporizer setting was decreased by 0.25%. If the dog responded to surgical stimulation, the vaporizer setting was increased by 0.5% and surgical manipulation was stopped until the dog reached an adequate plane of anaesthesia for surgery to continue.

Dogs were evaluated continuously during the recovery period by three investigators (veterinarians) blinded to the treatment group. The veterinarians assessing the dog’s recovery quality, comfort and pain had several years of experience assessing comfort and pain in dogs. The assessments were scored at: extubation, 15, 30, 60, 90, 120, and 180 minutes post-extubation. Each investigator assessed each dog individually to avoid any bias or external influence and the scores were averaged for analysis. Two pain scoring systems were used. A 0 to 10 visual analogue pain score (VAS), where 0 was considered to be comfortable with no pain and 10 was considered to be the worst possible pain or poor recovery from anaesthesia [17–18]. The second pain assessment used was the CSU canine acute pain scale with range 0 to 4 based on described observational behaviours and physical examination findings. Following extubation, recovery was considered painful when the dog vocalized, cried or responded to palpation of the surgical site. At later time points, the dogs were first observed in their kennel to assess their mentation, posture, facial expression, activity and behaviour. Then each evaluator interacted with the dog using a gentle approach to assess the dog’s attitude, movements and behaviour. If the dog appeared comfortable, the surgical site was gently palpated. If the dog remained comfortable, the dog was encouraged to stand up and take a few steps to fully assess pain status.

During the recovery period, if a dog was considered uncomfortable or painful having a VAS score above 3 or a CSU acute pain scale above 1.5, a rescue analgesia dose of morphine (0.1 mg/kg IV) was administered. If the dog remained painful, similar morphine rescue analgesia doses were repeated until comfort was achieved. At the end of the study period (180 min), carprofen (4 mg/kg SQ) was administered to all dogs for additional analgesia and morphine IV or SQ was administered as needed to maintain adequate analgesia and comfort.

Fifteen minutes following extubation, dogs were offered food (both canned and dry Purina EN^™^, Nestle Purina) and then again at 30, 60, 90, 120 and 180 minutes. The percentage of dogs that ate was recorded and compared between groups. All dogs were discharged to their owners the following day.

### Statistical analysis

Data distribution was assessed using (JMP Pro 11; SAS Institute Inc). Normally distributed data were compared using two tail unpaired t-test (body weight, propofol dose, HR, SAP, VAS score and CSU acute pain score). Data that were not normally distributed was compared using Mann-Whitney test (age, anaesthesia duration, surgery duration, RR, expired isoflurane concentration and post-operative appetite). Statistical significance difference between groups was considered when *p≤0*.*05* and statistical comparisons were performed using a statistical software (GraphPad, Prism 4.00 Software). A Pearson correlation coefficient was calculated between the 3 investigators who assessed postoperative pain (GraphPad, Prism 4.00 Software).

## Results

Thirty healthy intact female dogs between the age of 6 months and 6 years were enrolled in the study. Multiple purebred and mixed-breed dogs were included (S1 Appendix). Thirteen dogs received morphine and 17 dogs received maropitant. There was a difference in body weight between groups with the maropitant group being smaller (Table 1).

**Table 1:** Demographic data, anesthesia & surgery duration and propofol anesthesia induction dose used.

	Morphine	Maropitant	p
Age (months)	26 ± 22	16 ± 16	0.17
BW (kg)	20.8 ± 8	13.6 ± 7	0.02*
Anesthesia Duration (minute)	159 ± 34	136 ± 29	0.06
Surgery Duration (minute)	109 ± 31	89 ± 24	0.06
Propofol (mg/kg)	5.9 ± 1.7	6.8 ± 2.2	0.23

Data is presented as mean ± SD.

* Significant values p≤0.05.

Following pre-anaesthetic administration and prior to anaesthesia induction, the maropitant group subjectively did not show signs of sedation. The morphine group had a varied response ranging from no obvious sedation to heavy sedation, sleeping and ignoring their surroundings. The propofol induction dose required was similar between groups (Table 1). There was also no difference between total anaesthesia and surgery duration time between groups, however, these times approached significance (Table 1).

**Fig 1:**
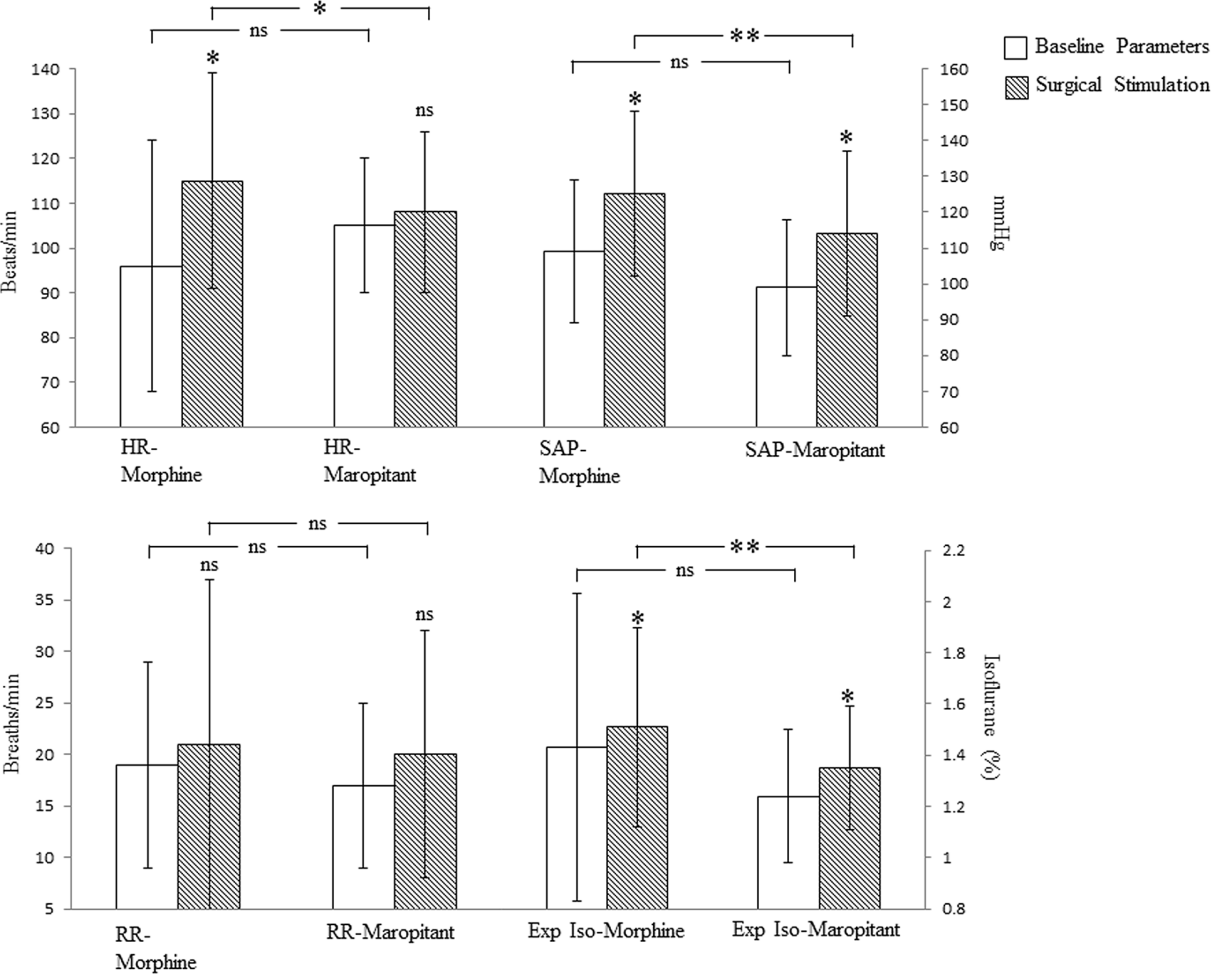
Physiologic parameters and expired isoflurane anesthesia concentration prior to and during ovariohysterectomy in dogs pre-medicated with morphine or maropitant. White bars are the baseline parameters obtained prior to surgery. Dashed bars are the averaged parameters obtained during OHE. HR = heart rate, SAP = systolic arterial blood pressure, RR = respiratory rate, Exp Iso = expired isoflurane concentration. Data is presented as mean ± SD. Significant values p≤0.05 & 0.01are depicted with * & ** respectively. Comparison between baseline parameters (white bars) and values obtained during OHE surgical stimulation (dashed bars) are depicted on top of the dashed bars. All other comparisons are indicated within the brackets.

The baseline physiologic parameters (HR, SAP, RR) and expired isoflurane concentration recorded during anaesthesia but before surgery were not different between groups (Fig 1).

During surgical stimulation the morphine group showed increased HR and SAP. The RR did not change (Fig 1). During the same time period of surgical stimulation, the maropitant group showed an increase in SAP but not in HR or RR. The SAP increase during surgical stimulation in the maropitant group was less than that of the morphine group (Fig 1). Similarly, the increase in HR in the morphine group was higher than the increase in HR in the maropitant group during surgical stimulation (Fig 1). RR had a large variability within each group, thus, neither group showed significant changes during surgical stimulation.

The expired isoflurane concentration increased significantly during surgical stimulation in both groups (Fig 1). However, the expired isoflurane changes during surgical stimulation were small with 9% and 6% increase for the maropitant and morphine groups respectively. The expired isoflurane during surgical stimulation was lower in the maropitant group (Fig 1).

At extubation, the maropitant group had lower VAS and CSU scores when compared to the morphine group (Table 2). During later time points (15, 30, 60, 90, 120 & 180 minutes post-extubation) there was no consistent difference observed between groups, when using either the VAS or CSU score systems (Table 2). The results from 15 minutes onward are likely confounded by the use of morphine rescue analgesia. During the recovery period, 13/17 and 10/13 of the dogs in the maropitant and morphine groups respectively required morphine rescue analgesia. The VAS and CSU assessment correlation between the 3 blinded investigators was considered good with r^2^ ≥ 0.76 and 0.72 respectively.

**Table 2:** Visual analogue pain score (VAS) and CSU acute pain scale from extubation to 180 minutes in the recovery period.

	Morphine	Maropitant	p value
VAS at extubation	4 ± 2	1.8 ± 1	0.0005**
CSU score extub	2.1 ± 0.8	1.4 ± 0.7	0.02*
VAS 15 minutes	2.9 ± 1.3	2.8 ± 1	0.69
CSU score 15	1.8 ± 0.6	1.9 ± 0.7	0.61
VAS 30 minutes	2.8 ± 0.9	2.4 ± 0.9	0.51
CSU score 30	1.8 ± 0.5	1.6 ± 0.5	0.24
VAS 60 minutes	2.6 ± 1	2.6 ± 0.8	0.9
CSU score 60	1.6 ± 0.5	1.6 ± 0.6	0.99
VAS 90 minutes	2.5 ± 1	2.6 ± 1.2	0.81
CSU score 90	1.5 ± 0.5	1.5 ± 0.6	0.83
VAS 120 minutes	2.2 ± 1.2	2.2 ± 1.1	0.47
CSU score 120	1.4 ± 0.7	1.3 ± 0.6	0.59
VAS 180 minutes	1.8 ± 0.9	1.5 ± 0.6	0.13
CSU score 180	1.3 ± 0.6	0.9 ± 0.4	0.04*

Data presented as mean ± SD.

* Significant values p≤0.05.

** Significant values p≤0.01.

Dogs were offered food beginning at the 15 minute postoperative time period. Between 15 and 180 minutes post-extubation period; 64.7% of the dogs in the maropitant group, while only 15.3% of the dogs in the morphine group ate any food (p = 0.02).

## Discussion

In the present study, the parameters recorded showed similarities between both groups, although dogs in the maropitant group had lower HR, SAP and required slightly lower isoflurane anaesthetic concentration. During recovery, at the extubation time point, the maropitant group had lower VAS and CSU scores. Dogs in the maropitant group were also more likely to eat during the first 180 minute of recovery period when compared to the morphine group. To our knowledge, this is the first report to describe the potential effects of maropitant for OHE surgeries in dogs. However, from an anaesthesia perspective, the overall difference between groups was small and further studies are necessary to identify the true clinical relevance of maropitant (NK-1 receptor antagonist) as a pre-anaesthetic agent for OHE surgery in dogs.

As a clinical trial, the study was influenced by inherent bias: the dogs were not evenly distributed between groups. Due to the randomization, fewer dogs were entered into the morphine group (13 compared to 17 in the maropitant group). The uneven distribution between the groups could predispose to a type II statistical error decreasing the study power and minimizing differences between groups. Dogs in the morphine group had a higher body weight when compared to dogs in the maropitant group. The difference in body weight could suggest either an age difference or a difference in the breed distribution between groups in the study. The blood pressure monitored and reported is only the SAP obtained via indirect ultrasonic doppler flow probe. Ideal blood pressure monitoring for research purposes would have been direct arterial pressure. Due to the invasiveness of direct arterial blood pressure monitoring, we considered that blood pressure measurement via indirect doppler was adequate for this clinical trial. The anaesthesia and surgery durations were not statistically different between the morphine and maropitant groups; however the difference was enough that it could have played a role.

Previous studies suggest that clinical trials and pain studies performed using canine OHE as a model are very difficult to interpret and distinct differences may not be readily observed between groups [18]. At extubation, we were not able to differentiate between pain, dysphoria or delirium as previously described in dogs recovering from anesthesia [19–21]. We considered any sign of poor recovery (high VAS or CSU score) to ethically treat and prevent any dog from being painful. Whether the dogs in the morphine group were more painful or had a higher incidence of poor recovery from anesthesia due to dysphoria or delirium at extubation remains to be studied further. Pain evaluation during the recovery period (15 to 180 minutes) was confounded by the use of morphine to manage postoperative pain. Morphine given to dogs can cause side effects such as nausea, vomiting, sedation, dysphoria or delirium. It is possible that these side effects could have also influenced the pain evaluation scores. The VAS and CSU assessment correlation between the 3 veterinarians blinded to the treatment group was considered to be good. Despite the subjectivity of the assessment; the correlation suggests a good degree of agreement between experienced veterinarians. The 3 veterinarians scoring the dogs considered the VAS system to have more flexibility to better assess the dog’s comfort and quality of recovery. VAS scoring has been previously used to assess the quality of recovery from anaesthesia in dogs [17,18]. The veterinarians felt subjectively that the CSU acute pain scale limited the scores to the clinical signs only described in the scale. For example, the CSU scale does not take into account sedation, when recovering from anaesthesia or when using rescue analgesia.

The physiological and pharmacological reasons for why maropitant, an NK-1 antagonist, may be adequate as a pre-anaesthetic agent for canine OHE remains to be elucidated. Maropitant is a potent antiemetic and decreasing post-operative nausea may be part of the explanation. Nausea could be an important clinical sign in canine post-operative pain. Another hypothesis is the potential ability of maropitant to decrease visceral pain. Visceral pain is defined as pain arising from the abdominal and thoracic organs. Visceral pain pathways travel to areas in the brain involved in pain perception. Some of these pathways such as the dorsal root ganglia, spinal cord dorsal horn, spinal cord ascending projections and brain structures considered important for nociception and pain processing; contain NK-1 receptors and substance P [9,22–29]. Nevertheless, the specific site of action for NK-1 receptor antagonists such as maropitant requires further investigation. A study looking at the effect of maropitant injected into the epidural space during ovarian ligament stimulation in dogs, showed no anaesthetic sparing effect [30]. On the other hand, the use of maropitant 1 mg/kg IV decreased the sevoflurane MAC in dogs by 24% in the same visceral pain model [14]. If we compare the maropitant 24% MAC sparing effect to morphine 1 mg/kg IV which decreases the halothane and isoflurane MAC in dogs by 30–40% [31,32]. It opens the question as to whether maropitant may have anaesthetic sparing activity at the level of higher CNS structures. On the contrary, a pancreatitis rodent model demonstrated that the NK-1 anti-nociception effect could be attributed to peripheral effects during pancreatitis [33]. Thus, further studies are necessary to fully understand the role of NK-1 antagonists and their site of action.

During the recovery period a greater percentage of dogs in the maropitant group ate, when offered food compared to the morphine group. The reason for increased interest in food during recovery it is unknown. There are many factors that could influence appetite in these patients such as environmental factors, post-operative anxiety, type and presentation of food, amount and frequency of rescue drug given for pain, or the continued presence of nausea or pain. None-the-less the postoperative period and environment was similar between groups and the maropitant treated dogs had a distinctly greater interest in food. The only information available regarding this effect suggests that maropitant prevents the incidence of vomiting when dogs are pre-medicated with hydromorphone for anaesthesia [34]. To our knowledge no other information is available regarding the use of maropitant for the perioperative period. Thus, additional studies investigating the maropitant’s role in post-operative nausea and appetite are required.

In the present clinical trial most dogs, independently of receiving maropitant or morphine required post-operative rescue analgesia. Thus, neither maropitant 1 mg/kg nor morphine 0.5 mg/kg alone appeared to be suitable for canine OHE, when surgery was performed by inexperienced personnel.

In summary, this study suggests that maropitant may minimize the HR and SAP response to surgical stimulation, while maintaining slightly lower isoflurane anaesthesia requirements, when compared to dogs given morphine as a pre-anaesthetic. The initial recovery quality also appears to be better and dogs are more likely to eat within the 3 hour post recovery period when maropitant is used as a pre-anaesthetic. However, when considering all measured parameters, no major clinical difference was observed between groups; suggesting that maropitant given as a pre-anaesthetic may be comparable to 0.5 mg/kg of morphine for an OHE and may improve the post-operative recovery.

## Supporting Information

S1 Appendix Breed distribution between treatment groups. (DOCX)
