# Supplementary material for: Comparison of NK-1 Receptor Antagonist (Maropitant) to Morphine as a Pre-Anaesthetic Agent for Canine Ovariohysterectomy
Source: PLoS One. 2015 Oct 29;10(10):e0140734. doi: 10.1371/journal.pone.0140734 (PMC4626099; doi:10.1371/journal.pone.0140734)
Supplement: S1 Appendix — (DOCX) [file pone.0140734.s001.docx]

**S1-Appendix 1.** Breed distribution between treatment groups.

| Morphine | Maropitant |
| --- | --- |
| Boston Terrier 1  German Shorthair Pointer 1  Soft Coated Wheaton Terrier 1  Pit Bull Terrier 2  Labrador Retriever 2  Chesapeake Bay Retriever 1  Australian Shepherd 1  Australian Cattle Dog 1  Jack Russell Terrier 1  Weimaraner 1  Mixed breed 1 | Labrador Retriever 3  Boston Terrier 1  Border Collie 2  Pit Bull Terrier 1  Min Schnauzer 1  Siberian Husky 1  Jack RussellTerrier 1  Pug 1  West Highland White Terrier 1  German Shepherd 1  Australian Shepherd 1  Rottweiler 2  Mixed breed 1 |
